# Decreased sirtuin 1 in type 2 diabetes patients with abnormal BMD

**DOI:** 10.3389/fendo.2024.1480847

**Published:** 2025-01-29

**Authors:** Yao Xu, Tianxiao Hu, Peiwu Jiang, Xiujing Wang, Jiaqi Yao, Huiling Shen, Zhenying Zhang, Bojing Zheng, Ting Wang, Yanxia Ren, Jing Wang, Qingying Tan

**Affiliations:** ^1^ Department of Endocrinology, No.903 Hospital of PLA Joint Logistic Support Force, Hangzhou, China; ^2^ Department of Vascular Surgery, Hangzhou TCM Hospital Affiliated to Zhejiang Chinese Medical University, Hangzhou, China

**Keywords:** Sirtuin 1, bone mineral density, bone turnover markers, type 2 diabetes mellitus, bone metabolism

## Abstract

**Introduction:**

Sirtuin 1, a class III histone deacetylase, plays a critical role in the pathophysiology of both diabetes mellitus and bone metabolism by promoting osteoblast differentiation and inhibiting osteoclast maturation. However, its exact impact on bone mineral density (BMD) and bone metabolism in type 2 diabetes mellitus (T2DM) remains unclear. This study investigates the relationship between Sirtuin 1 levels, BMD, and bone metabolism in newly diagnosed T2DM patients, specifically examining alterations in Sirtuin 1 levels in those with concomitant osteoporosis or osteopenia.

**Methods:**

A total of 69 newly diagnosed T2DM patients and 82 control subjects with normal glucose tolerance (NGT) were enrolled. Serum Sirtuin 1 levels and bone turnover markers, including osteocalcin (OC), procollagen type 1 N-terminal propeptide (P1NP), and β-C-terminal telopeptide of type I collagen (β-CTX), were measured using enzyme-linked immunosorbent assay (ELISA). BMD was assessed via dual-energy X-ray absorptiometry (DXA). Comparisons of these parameters were made between the T2DM and NGT groups.

**Results:**

T2DM patients were further categorized into a normal BMD group (DMn) and an osteopenia or osteoporosis group (DMo), and differences in Sirtuin 1 levels between these subgroups were analyzed. Risk factors for osteoporosis/osteopenia in T2DM patients were also evaluated. Serum Sirtuin 1 levels were found to be significantly diminished in the T2DM group relative to the control group (P < 0.05), with no significant differences in lumbar spine BMD, OC, 25(OH)D, and β-CTX between groups (P > 0.05). Osteoporosis incidence was higher in T2DM subjects compared to controls (34.8% vs. 18.3%, P = 0.026). Subgroup analysis revealed that SIRT1 levels in T2DM patients with osteoporosis or osteopenia exhibited a significant reduction compared to those with normal BMD (P < 0.05). Logistic regression indicated that Sirtuin 1, age, HDL-C, P1NP, and β-CTX were independent risk factors for osteoporosis in T2DM patients.

**Discussion:**

In conclusion, decreased serum Sirtuin 1 levels are associated with bone turnover markers in T2DM patients and may serve as an independent risk factor and potential biomarker for diagnosing bone metabolism disorders in newly diagnosed T2DM patients.

## 1 Background

Type 2 diabetes mellitus (T2DM) is associated with an elevated risk of fractures and increased post-fracture mortality. Despite having higher bone mineral density (BMD), individuals with T2DM face a paradoxically higher risk of fractures, complicating the prediction and management of fracture risk in this population (1). Hyperglycemia contributes to increased bone matrix rigidity and reduced bone toughness by promoting the accumulation of advanced glycation end products (AGEs) (2). Additionally, insulin resistance or insufficient insulin secretion further suppresses osteoblast function, leading to decreased bone formation (3). Furthermore, patients with T2DM often exhibit a state of chronic low-grade inflammation, characterized by elevated levels of inflammatory cytokines such as tumor necrosis factor-alpha (TNF-α) and interleukin-6 (IL-6). These cytokines disrupt bone metabolic balance by enhancing osteoclast activity and inhibiting osteoblast differentiation (4). Simultaneously, oxidative stress and microvascular complications indirectly impair bone health by damaging osteocytes and reducing bone blood supply (5).

Commonly utilized clinical bone turnover markers, such as type 1 collagen amino-terminal propeptide (P1NP) and carboxy-terminal collagen crosslinks (CTX), effectively reflect bone turnover changes and predict fracture risk in non-diabetic individuals (6). However, these markers do not correlate with fracture risk in T2DM patients, thus limiting their utility in this context. Furthermore, there is a significant gap in the understanding of the molecular mechanisms that underlie changes in bone density during the progression of T2DM.

Sirtuin 1 (SIRT1), a class III histone deacetylase, is known for its extensive biological activities, primarily through the modulation of various transcription factors such as p53, FoxOs, NF-κB, and PPARγ, all of which play critical roles in multiple disease processes (7). In the context of diabetes mellitus, SIRT1 serves as a protective factor for pancreatic β-cells, enhancing insulin secretion in response to glucose, promoting gluconeogenesis, reducing oxidative stress, and ameliorating insulin resistance in key metabolic tissues such as the liver, adipose tissue, and skeletal muscle (8, 9). Studies focusing on bone metabolism have revealed that SIRT1 plays a role in promoting osteoblast differentiation while inhibiting osteoclast maturation, In the molecular regulation of bone metabolism, SIRT1 plays a critical protective role by promoting osteoblast differentiation through activation of the Wnt/β-catenin signaling pathway. SIRT1 also suppresses osteoclast maturation and reduces the production of inflammatory cytokines, contributing significantly to the regulation of bone metabolism. However, its precise role in bone density and metabolism within the context of type 2 diabetes mellitus (T2DM) pathogenesis remains poorly understood (10). This study offers an initial investigation into the potential involvement of Sirtuin 1 in alterations of bone density among patients with T2DM, and its potential utility as a biomarker for evaluating bone metabolism. Through this exploration, the study aims to contribute clinical evidence towards identifying novel biomarkers for assessing bone metabolism in individuals with T2DM.

## 2 Materials and methods

### 2.1 Research objects

The study was approved by the Ethics Committee of Chinese 903rd Hospital of PLA, conducted in accordance with the Declaration of Helsinki. Prior to this study, written informed consent was obtained from all the subjects. Data were collected from both outpatients and inpatients at the Department of Endocrinology, 903rd Hospital of PLA, China, between March 2015 and August 2023.

According to the inclusion and exclusion criteria of the 1999 World Health Organization (WHO) criteria for diabetes diagnosis and classification, the specific method for the oral glucose tolerance test involves administering a solution containing 75 grams of anhydrous glucose to adults, a total of 69 newly diagnosed T2DM patients were included as the T2DM group (mean age 48 years)and 82 patients were carried as the NGT group(mean age 49 years). Then divide into four subgroups on the basis of BMD levels [normal BMD subjects (−1.0 < T/Z < 1.0) and decreased BMD subjects (T/Z ≤ −1.0, including osteopenia and osteoporosis)] for further analysis. Exclude type 1 diabetes patients and type 2 diabetes patients who have used hypoglycemic drugs, chronic renal insufficiency, history of parathyroid or thyroid diseases, with a fracture history, long-term use of glucocorticoids, heparin, antiepileptic drugs, antipsychotic medications, thiazides, calcium supplements, or other medications affecting bone metabolism and anti-osteoporotic drugs.

### 2.2 Anthropometric and biochemical parameters

Anthropometric measurements were conducted with high precision following standardized methodologies. Height was recorded in a standing position using a wall-mounted stadiometer (VM Electronics, Chicago, IL, USA), while body weight was obtained to the nearest 0.1 kg with a digital scale. Hip circumference was accurately measured at the maximal protrusion of the gluteal region, and waist circumference (WC) was precisely determined at the widest point between the xiphoid process and the iliac crest. Key indices of adiposity, including waist-to-hip ratio (WHR) and body mass index (BMI), were calculated with rigorous attention to detail. BMI was derived by dividing weight (kg) by height squared (m²), and WHR was calculated as the ratio of waist circumference (cm) to hip circumference (cm), both important metrics for assessing body composition. After anthropometric assessments, blood pressure measurements, including systolic (SBP) and diastolic blood pressure (DBP), were obtained under standardized conditions.

Blood samples were collected after a minimum 10-hour overnight fast and stored at -80°C for subsequent analysis. Advanced biochemical parameters were measured using an automatic biochemical analyzer (Abbott, Aeroset C16000, USA) for accurate quantification of total triglycerides (TG), total cholesterol (TC), low-density lipoprotein cholesterol (LDL-C), high-density lipoprotein cholesterol (HDL-C), fasting blood glucose (FBG), and 2-hour plasma glucose (2hPG). Fasting insulin (FINS) was measured via a chemiluminescence immunoassay analyzer (Abbott, I4000, USA), and glycosylated hemoglobin (HbA1c) was assessed using the Afinion AS100 analyzer (Alere, Afinion AS100, USA). Additionally, insulin resistance (HOMA-IR) and β-cell function (HOMA-β) were rigorously calculated using the homeostasis model assessment (HOMA) equations: HOMA-IR = FBG (mmol/L) × FINS (μU/mL)/22.5, and HOMA-β = 20 × FINS (μU/mL)/[FBG (mmol/L) − 3.5].

### 2.3 Measurements of Sirtuin 1, bone turnover markers, and BMD

Enzyme-linked immunosorbent assays (ELISAs) were utilized for the quantification of sirtuin 1 levels (Dingguo, Guangzhou, China), expressed in umol/L. Serum levels of osteocalcin (OC), procollagen type 1 intact N-terminal propeptide (P1NP), β-C-terminal telopeptide of type I collagen (β-CTX), and 25-hydroxyvitamin D (25(OH)D) were determined via chemiluminescence immunoassay (CLIA) kits following the manufacturers’ instructions (Beckman, USA). Bone mineral density (BMD) of the L1–L4 vertebrae (Lumbar BMD) was assessed using dual-energy X-ray absorptiometry (DXA) (GE Medical systems lunar, USA) and expressed as absolute values (g/cm2), T-scores, or Z-scores.

### 2.4 Statistical analysis

The continuous variables with a normal distribution were expressed as means ± standard deviation (SD) and analyzed using independent samples t-test. For variables deviating from a normal distribution, data were presented as median [interquartile range (IQR)] and analyzed using the Mann–Whitney test. Subgroup comparisons were conducted using one-way ANOVA followed by *post hoc* analysis. Pearson correlation analysis was applied to assess the association between sirtuin1 levels and parametric variables, while Spearman correlation analysis was used for nonparametric variables. Binary logistic regression analysis was utilized to determine risk factors for osteoporosis. A significance level of p < 0.05 was considered statistically significant. All statistical analyses were performed using SPSS version 29.0 software.

## 3 Results

### 3.1 The clinical parameters and serum SIRT1 levels of the two groups

**Figure 1:**
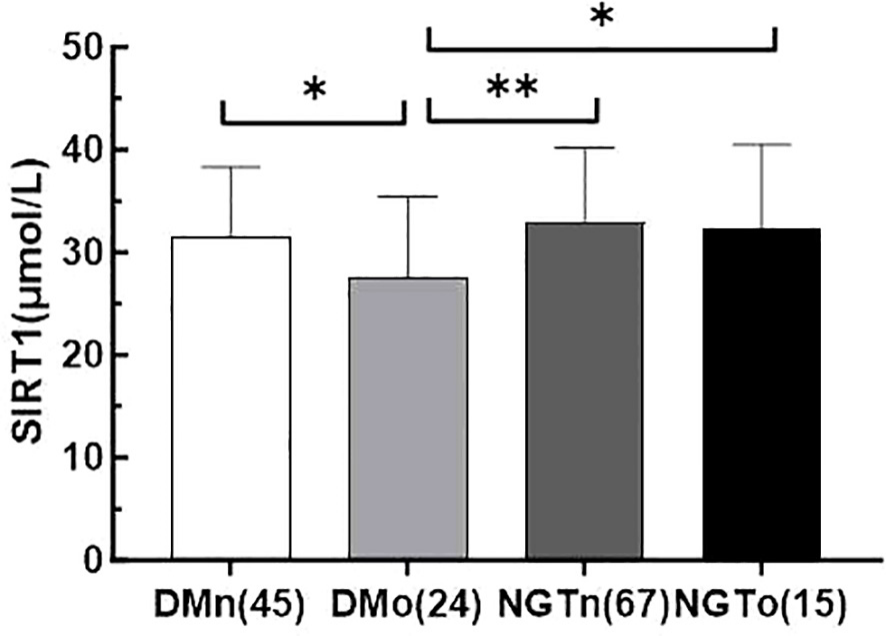
Serum irisin levels in four subgroups of DMn (T2DM with normal BMD), DMo (T2DM with osteopenia or osteoporosis), NGTn (NGT with normal BMD), and NGTo (NGT with osteopenia or osteoporosis). The number of subjects was shown in parentheses. values are evaluated by a Tukey (HSD) test. * indicate P< 0.05, **  indicate P < 0.01.

All collected data are summarized in **Tables 1** and **2**. Compared to the control subjects, patients with T2DM exhibited significantly higher diastolic blood pressure (DBP), weight, BMI, waist circumference, triglycerides (TG), and total cholesterol (TC), alongside lower levels of procollagen type 1 N-terminal propeptide (PINP) and high-density lipoprotein cholesterol (HDL-C) (P < 0.01 or P < 0.05). Notably, serum SIRT1 levels in the T2DM group were markedly reduced than those in the control group (P < 0.05).No significant differences were observed between the two groups regarding lumbar BMD, osteocalcin (OC), 25-hydroxyvitamin D [25(OH)D], and β-C-terminal telopeptide of type I collagen (β-CTX) (P > 0.05). The incidence of osteoporosis was higher in the T2DM group compared to the control group (34.8% vs. 18.3%, P = 0.026). Furthermore, SIRT1 levels in T2DM patients with osteoporosis (DMo) were substantially lower than in NGT patients with osteopenia or osteoporosis (NGTo) (27.50 ± 7.95 vs. 32.34 ± 8.18 μmol/L, P < 0.05).However, no significant difference in serum SIRT1 levels was found between NGT patients with osteopenia or osteoporosis (NGTo) and those with normal BMD (NGTn) (32.34 ± 8.18 vs. 32.90 ± 7.32 μmol/L, P > 0.05), as illustrated in **Figure 1**.

**Table 1:** Clinical physical parameters of controls and T2DM subjects.

	NGT (n=82)	T2DM (n=69)	t/χ^2^/Z	P
Gender,M/F	43/39	45/24	-1.581	0.114
Age(years)	49.00(43.75,55.00)	48.00(43.00,56.00)	-0.178	0.859
SBP(mmHg)	124.00(112.00,135.25)	125.00(120.00,140.00)	-1.799	0.072
DBP(mmHg)	76.43 ± 10.45	82.67 ± 9.95	3.740	<0.001*
Weight(kg)	65.70 ± 11.74	70.64 ± 13.50	2.408	0.017*
Height(cm)	164.75(159.75,170.00)	167.00(160.00,170.25)	-0.209	0.834
BMI(kg/m^2^)	24.04 ± 3.06	25.96 ± 4.32	3.186	0.002*
Waist circumference(cm)	83.59 ± 9.87	89.54 ± 11.45	3.430	<0.001*
Hip circumference(cm)	93.00(89.00,97.25)	94.00(89.00,97.00)	-0.284	0.776
WHR	0.90(0.84,0.95)	0.95(0.91,1.00)	-4.534	<0.001*

Values were given as means ± SD or median with interquartile range. NGT, normal glucose tolerance; T2DM, type 2 diabetes mellitus; SBP, systolic blood pressure; DBP, diastolic blood pressure; BMI, body mass index; WHR, waist-hip ratio. *P<0.05.

**Table 2:** serum test parameters of controls and T2DM subjects.

	NGT (n=82)	T2DM (n=69)	t/χ^2^/Z	P
HbAlc (%)	5.40 (5.28,5.60)	7.50 (6.30,10.20)	-9.907	<0.001*
FPG (mmol/L)	4.86 (4.47,5.29)	7.48 (5.94,11.07)	-9.193	<0.001*
2hPG (mmol/L)	6.14 (5.01,6.87)	13.88 (11.81,17.86)	-10.385	<0.001*
FINS (uIU/ml)	5.92 (4.30,8.83)	8.70 (5.70,11.95)	-23.114	0.002*
HOMA-IR	1.28 (0.94,1.98)	3.08 (1.80,5.49)	-6.539	<0.001*
HOMA-β	93.63 (60.46,153.01)	46.15 (23.23,79.98)	-5.999	<0.001*
TC (mmol/L)	4.61 (4.09,5.30)	5.11 (4.48,5.95)	-2.942	0.003*
TG (mmol/L)	1.17 (0.90,1.98)	2.01 (1.33,3.43)	-4.606	<0.001*
LDL-C (mmol/L)	2.67 ± 0.72	2.72 ± 1.04	0.443	0.659
HDL-C (mmol/L)	1.26 (1.11,1.49)	1.06 (0.95,1.2)	-4.131	<0.001*
25 (OH)D (ng/ml)	20.33 (15.49,25.53)	18.47 (14.79,23.45)	-0.966	0.334
Lumbar BMD (g/cm^2^)	1.11 ± 0.13	1.10 ± 0.15	-0.465	0.643
P1NP (ng/ml)	43.75 (35.17,60.97)	38.06 (29.56,54.41)	-2.107	0.035*
OC (ng/ml)	18.35 (15.47,24.12)	17.21 (13.59,21.05)	-1.885	0.059
β-CTX (pg/L)	470 (310,660)	430 (270,600)	-0.443	0.658
SIRT1 (μmol/L)	32.80 ± 7.43	30.08 ± 7.44	-2.244	0.026*

Values were given as means ± SD or median with interquartile range. HbA1c, glycosylated hemoglobin A1c; FBG, fasting plasma glucose; 2hPG, 2-hour plasma glucose; FIN, fasting insulin; HOMA-IR, homeostasis model assessment of insulin resistance; HOMA-β, homeostasis model assessment of β-cell function; TC, total cholesterol; TG, triglyceride, LDL-C, low-density lipoprotein; HDL-C, high-density lipoprotein; Lumbar BMD, body mineral density of the L1–L4 vertebrae. P1NP, procollagen type 1 intact N-terminal propeptide; OC, osteocalcin; β-CTX, β-C-terminal telopeptides of type I collagen. *P<0.05.

### 3.2 Relevant factors in pathogenesis of osteoporosis

In the NGT group, lumbar BMD was found to have a significant negative correlation with PINP (r = -0.236, P < 0.05). In contrast, in the T2DM group, lumbar BMD was inversely correlated with age (r = -0.313, P < 0.01). Additionally, lumbar BMD in the T2DM group showed positive correlations with waist circumference (r = 0.279, P < 0.05), weight (r = 0.333, P < 0.01), height (r = 0.272, P < 0.01), and hip circumference (r = 0.245, P < 0.05) (**Table 3**), correlations that were not observed in the NGT group. Stepwise binary logistic regression analysis further suggested that SIRT1, age, HDL-C, PINP, and β-CTX were independently associated with the pathogenesis of osteoporosis (P < 0.01 or P < 0.05) (**Table 4**).

**Table 3:** Correlations of BMD with clinical characteristics in the two groups.

		NGT r P		T2DM r P	
Lumbar BMD					
vs	Age(years)	-0.173	0.121	-0.313	0.009*
vs	Weight(kg)	0.056	0.616	0.333	0.005*
vs	Height(cm)	0.072	0.510	0.272	<0.001*
vs	Waist circumference(cm)	0.106	0.342	0.279	0.020*
vs	Hip circumference(cm)	0.112	0.317	0.245	0.042*
vs	25(OH)D(ng/ml)	-0.179	0.107	-0.033	0.789
vs	P1NP(ng/ml)	-0.236	0.033*	0.012	0.920
vs	OC(ng/ml)	-0.207	0.063	-0.150	0.218
vs	β-CTX(ng/L)	-0.167	0.134	-0.231	0.057

BMD, bone mineral density; NGT, normal glucose tolerance; T2DM, type 2 diabetes mellitus; P1NP, procollagen type 1 N-terminal propeptide; β-CTX, β-C-terminal telopeptide of type I collagen; OC, osteocalcin; 25(OH)D, 25-hydroxyvitamin D. *P<0.05.

**Table 4:** Binary logistic regression analysis of risk factors for developing osteoporosis in T2DM group.

Variables	β Coefficient	OR(95% CI)	P-value
Age (years)	0.224	1.251 (1.030-1.518)	0.024
HDL-C (mmol/L)	-5.815	0.003 (0.000-0.568)	0.030
P1NP (ng/ml)	-0.163	0.849 (0.730-0.998)	0.034
β-CTX (pg/L)	0.009	1.010 (1.002-1.017)	0.013
SIRT1 (μmol/L)	-0.239	0.787 (0.644-0.962)	0.019

P1NP, procollagen type 1 N-terminal propeptide; HDL-C, high-density lipoprotein cholesterol; β-CTX, β-C-terminal telopeptide of type I collagen; SIRT1, Sirtuin 1.

## 4 Discussion

Sirtuin 1 is a deacetylase enzyme with extensive biological functions, capable of targeting and regulating numerous post-translational regulatory factors, including p53, FoxOs, nuclear transcriptional regulator NF-κB, and peroxisome proliferator-activated receptor γ (PPARγ), and is associated with various diseases (11, 12). The role of Sirtuin 1 in diabetes is well-established; it acts as a protective factor for pancreatic β-cells and promotes glucose-dependent insulin release (13, 14). Additionally, it enhances gluconeogenesis, reduces oxidative stress, and improves insulin resistance in the liver, adipose tissue, and skeletal muscle. Studies on bone metabolism regulation have shown that Sirtuin 1 can promote osteogenic differentiation and inhibit osteoclast maturation through various mechanisms (15, 16). However, changes in Sirtuin 1 in patients with T2DM and abnormal bone mass remain unclear (17).

The findings of this study demonstrate that newly diagnosed T2DM patients exhibit lower serum levels of Sirtuin 1 and P1NP compared to the normal control group. Subgroup analysis further reveals that T2DM patients with osteoporosis (DMo) have significantly lower serum Sirtuin 1 levels compared to those with normal bone mineral density (DMn). Logistic regression analysis highlighted that reduced SIRT1 levels, alongside age, HDL-C, P1NP, and β-CTX, were independent risk factors for osteoporosis in T2DM patients. However, conflicting results exist regarding the expression of SIRT1 in diabetic patients (18). These discrepancies may be attributed to varying stages of diabetes and differences in disease severity. Research has shown that one month after the onset of diabetes in rats, SIRT1 expression in cardiac tissue increases, while after three months, SIRT1 levels decrease (18). Studies reporting decreased SIRT1 levels often involve cases with greater diabetes severity (19). Moreover, SIRT1 deficiency has been associated with increased oxidative stress, as well as progression of diabetic retinopathy and cognitive dysfunction (20, 21). In our study, all participants were newly diagnosed, untreated T2DM patients, with an HbA1c level of approximately 7.5%, indicating a moderate degree of disease severity (22).

The logistic regression results of this study indicate that hypercholesterolemia also serves as an independent protective factor against osteoporosis in diabetic patients (23). However, the impact of HDL-C on osteoporosis remains inconclusive (24). In the presence of HDL, osteoblast lysosomes are more likely to maintain their integrity. Increased expression of scavenger receptor class B type I (SR-BI) reduces oxLDL uptake and enhances selective cholesterol absorption, thereby regulating oxLDL concentration. Studies at the cellular level suggest that high HDL levels are beneficial for osteoblast survival, contributing to improved bone density. HDL-C can inhibit LDL-C oxidation and prevent the associated multi-organ functional damage, thus exhibiting a protective effect. However, whether HDL-C has a definitive protective role against osteoporosis warrants further investigation (25).

The results of this study indicate no significant difference in bone mineral density (BMD) between the diabetic and non-diabetic groups. However, the diabetic group exhibited a notable decline in SIRT1 levels compared to the non-diabetic group, suggesting that alterations in SIRT1 may precede BMD reductions as an indicator of bone metabolism abnormalities. Reports on BMD changes in T2DM patients are inconsistent, with some studies finding decreased BMD, while others report no change or even an increase. Given that bone mass changes in T2DM patients are influenced by various factors, the mechanisms of diabetes-induced osteoporosis are currently thought to be related to sex, age, measurement site, and BMI, as well as insulin deficiency or resistance, diabetes complications, and hyperlipidemia. However, reports on the effects of these factors on osteoporosis remain inconclusive. Using chi-square analysis, this study found a higher prevalence of abnormal bone mass in the diabetic group compared to the non-diabetic group. As BMD was used in the initial comparison between groups, and chi-square analysis was based on T-scores to determine bone mass abnormalities, the positive correlation between BMD and T-scores may contribute to the discrepancy between these results. The inconsistency may primarily stem from two factors: First, the sample size is relatively small. Second, due to the limited number of osteoporosis patients, we combined those with reduced bone mass and osteoporosis into a single abnormal bone mass group, resulting in a minimal BMD difference. This limitation should be noted as a shortcoming of the study.

## 5 Conclusion

This study demonstrates that serum Sirtuin 1 levels are significantly lower in T2DM patients compared to non-diabetic controls, with an even greater reduction observed in those with osteoporosis. Sirtuin 1 has been identified as an independent protective factor against osteoporosis in T2DM patients and may serve as an earlier indicator of bone metabolism abnormalities than declines in bone mineral density (BMD). Additionally, hypercholesterolemia appears as an independent protective factor against osteoporosis in diabetic patients. Although no difference in BMD was observed between diabetic and non-diabetic groups, the prevalence of abnormal bone mass was higher in diabetic patients. Further investigation into the mechanistic role of Sirtuin 1 and the protective effects of other related factors in diabetic osteoporosis may provide valuable insights into the pathogenesis of T2DM-associated bone metabolic abnormalities and inform early intervention strategies.

## Data Availability

The original contributions presented in the study are included in the article/supplementary material. Further inquiries can be directed to the corresponding author.
